# Broadband solar absorption enhancement via periodic nanostructuring of electrodes

**DOI:** 10.1038/srep02928

**Published:** 2013-10-14

**Authors:** Michael M. Adachi, André J. Labelle, Susanna M. Thon, Xinzheng Lan, Sjoerd Hoogland, Edward H. Sargent

**Affiliations:** 1 Department of Electrical and Computer Engineering, University of Toronto, Toronto, Ontario M5S 3G4, Canada; 2 School of Materials Science and Engineering, Hefei University of Technology, 193 Tunxi Rd, Hefei, Anhui Province, 230009, P. R. China; 3 Current address: Department of Electrical and Computer Engineering, Johns Hopkins University, Baltimore, Maryland 21218, USA.

## Abstract

Solution processed colloidal quantum dot (CQD) solar cells have great potential for large area low-cost photovoltaics. However, light utilization remains low mainly due to the tradeoff between small carrier transport lengths and longer infrared photon absorption lengths. Here, we demonstrate a bottom-illuminated periodic nanostructured CQD solar cell that enhances broadband absorption without compromising charge extraction efficiency of the device. We use finite difference time domain (FDTD) simulations to study the nanostructure for implementation in a realistic device and then build proof-of-concept nanostructured solar cells, which exhibit a broadband absorption enhancement over the wavelength range of λ = 600 to 1100 nm, leading to a 31% improvement in overall short-circuit current density compared to a planar device containing an approximately equal volume of active material. Remarkably, the improved current density is achieved using a light-absorber volume less than half that typically used in the best planar devices.

Colloidal quantum dot (CQD) solar cells have gained much interest in recent years in view of their potential for low cost manufacture via solution-coating, combined with their tunable bandgap^1^,^2^,^3^,^4^,^5^, which offers to maximize the spectral range of solar light that can be efficiently harvested. Nonetheless, much of the available solar spectrum in state-of-the-art CQD solar cells remains to be fully utilized, mainly due to limited charge-carrier transport lengths in CQD films^6^,^7^ which limit the maximum film thickness for carrier extraction to less than the absorption length for infrared photons in CQD films. As one example, the highest certified power conversion efficiency CQD solar cell (7%) showed a maximum external quantum efficiency of ~80% at short wavelengths (λ < 550 nm) which dropped to below 30% at longer wavelengths (λ > 800 nm)^5^.

As a result, photon management schemes such as plasmonic enhancement using gold nanoshells^8^, as well as multi-pass light management schemes^9^, are of interest for improving light absorption in CQD solar cells. In the field of thin film Si, a number of studies report on the use of sub-wavelength sized periodic structures such as 2D arrays of nanodomes/nanostructures for reducing reflectance and enhancing absorption by coupling to guided modes^10^,^11^ and arrays of nanowells for efficient light collection by optical diffraction^12^. Other examples of optical absorption enhancement schemes for thin films include, but are certainly not limited to: excitation of plasmonic modes in metallic gratings^13^,^14^,^15^,^16^,^17^, localized surface plasmons on metal nanoparticles^18^, light-trapping using distributed Bragg reflectors with a grating^19^,^20^ or 2D and 3D arrays of photonic crystals^21^, formation of whispering-gallery resonant modes in spheres^22^ and shells^23^, and enhancement by leaky-mode resonances in optical antennas^24^.

Periodic structuring for light trapping is of particular interest due to the opportunity to use well-developed fabrication methods and the ease of designing structures that take advantage of effects such as light localization and waveguiding. Such periodic structures are usually incorporated into top-illuminated architectures so that the metal back-reflector can be structured directly before depositing the light-absorbing active layers on top. Less attention has been put on bottom-illuminated structures; however, in the case of CQD solar cells, the best solar cell efficiency has been reported for a bottom-illuminated TiO_2_/PbS heterojunction architecture. Bottom-illuminated architectures are required to make the best CQD cells today since the electron-extracting metal oxide electrode relies on high-temperature processing.

In this work we therefore explored periodic bottom-illuminated sub-wavelength structures to enhance broadband absorption while building on the highest performing CQD solar cell architectures. This allowed us to increase appreciably the current density in a device having less than half the absorber volume used in CQD planar devices^5^,^8^,^25^. The mechanism of absorption enhancement is enhanced localized absorption near the metal/semiconductor interface attributed to surface plasmon polaritons and strong reduction in broadband reflection.

## Results

We required in our design that the collection distance (defined as the maximum distance any charge carrier would have to travel in the film to be collected at its selective electrode) of 200 nm should be maintained in order to maximize the device internal quantum efficiency. The design should be bottom illuminated in order to take advantage of fabrication procedures for the highest performing CQD solar cells to date^25^. In addition, unlike Si, which has a high refractive index of n = 3.6 at the wavelength, λ = 1 μm, CQD PbS films have a much lower index of ~2.6 at the same wavelength^8^; therefore to maximize the refractive index contrast for facilitating multiple internal reflections at the device interfaces, the glass should be structured and conformally coated by a thin indium tin oxide (ITO) bottom electrode. To maximize absorption while maintaining short transport lengths, the PbS CQD layer should conformally coat the TiO_2_ layer (50 nm in thickness).

**Figure 1:**
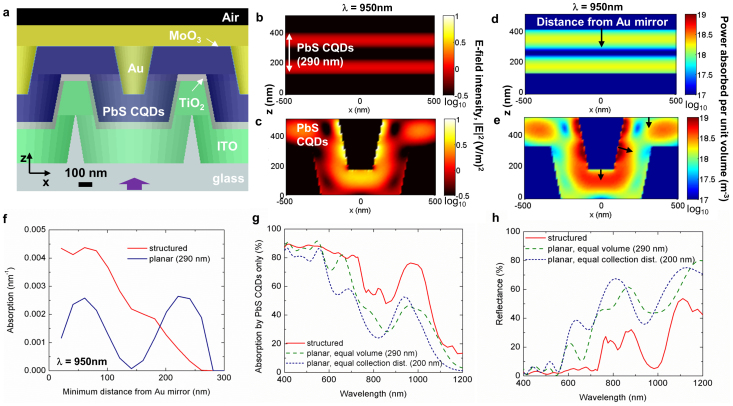
FDTD simulations of the proposed nanostructured colloidal quantum dot solar cell. (a) Cross section illustration of a 3D hexagonal array of the nanostructured CQD solar cell with an ideally conformal PbS CQD film (collection distance = 200 nm) and periodicity of 1 μm. (b) The electric field squared (|E|^2^) color plot of an equal volume (thickness = 290 nm) PbS CQD planar film and (c) nanostructured PbS CQD film at the exciton wavelength λ = 950 nm. (d) The power absorbed per unit volume color plot of the equal volume PbS CQD planar film and (e) nanostructured PbS CQD film at λ = 950 nm. Only the |E|^2^ and power absorbed per unit volume in the PbS region is plotted. (f) The integrated absorption in the PbS CQD film as a function of distance from the top Au mirror (Au) at λ = 950 nm. (g) The integrated absorption in the PbS CQD film and (h) calculated reflectance for an equal collection distance planar, equal volume planar, and nanostructured device.

In designing the exact periodic structure for enhancing the current, we needed to select feature heights, periods, and geometric shapes that would result in the largest broadband absorption enhancement in our spectral range of interest while being compatible with our sub-wavelength patterning process (i.e. nanosphere lithography) and CQD photovoltaic device fabrication processes. Another critical consideration was maintaining minimum collection distances for photo-generated carriers in the CQD film. Taking inspiration from recent work on light localization and antenna effects in nanostructured dielectric core-shell systems^24^, we arrived at the structure illustrated in Figure 1a. The top contact consists of a thin MoO_3_ layer with an optimal thickness of 5 nm^26^ and thick Au top electrode. The deep work function MoO_3_ layer is used to obtain high open-circuit voltage by preventing the formation of a voltage-limiting Schottky contact^26^. The structure is periodic in the x and y directions but offset from neighboring structures by an angle of 60°, forming a hexagonal array along the x-y plane. All layers shown in Figure 1a (Au, MoO_3_, PbS CQDs, TiO_2_, ITO, glass) were included in the simulations. The electric field squared (E^2^) color maps, calculated by FDTD simulation at the exciton wavelength λ = 950 nm, are shown in Figure 1b for a planar device containing an equal volume of the CQD active material as the structured device and Figure 1c for the nanostructured device illustrated in Figure 1a. For the planar device, the electric field intensity appears as an interference pattern between incident and reflected waves from the Au mirror. For the nanostructured device, enhancement in electric field intensity occurs along the edges of the Au contact in Figure 1c. The intensity enhancement near the surface of the Au is due to surface plasmon polaritons, or bound waves that travel along the metal/semiconductor interface^27^, in this case the Au/CQD interface. Localization of power absorbed per unit volume for the nanostructured substrate is shown in Figure 1e. To further demonstrate the localization of light along the Au/CQD interface, we show in Figure 1f the integrated absorption versus minimum distance from the Au mirror for every mesh point in the three-dimensional PbS CQD region at the exciton wavelength, λ = 950 nm. For the planar device, absorption maxima occur at 60 and 240 nm from the Au mirror. In contrast, absorption in the structured device peaks nearest the Au mirror and decreases steadily toward the TiO_2_ layer. Note that the area under the curves leads to total fractional absorption of 0.47 for the planar CQDs (thickness = 290 nm) and 0.75 for the structured CQDs (also shown in figure 1e at λ = 950 nm).

The full spectrum of absorption in the CQD layer is shown for each case (planar and structured) in Figure 1g. Three cases are considered: planar with thickness equal to the 200 nm collection distance; planar with thickness of 290 nm chosen to equalize CQD volume; and the structured case. All devices include a Au mirror. The structured PbS film shows strong broadband absorption enhancement compared to both planar cases, with the greatest enhancement occurring at long wavelengths (λ = 600 nm to 1200 nm) where the need for absorption enhancement is the greatest in view of the CQD film's weaker absorption per unit length. The anti-reflective properties of the nanostructured device are also shown in Figure 1h. The nanostructured substrate shows noticeably lower reflectance (R) than both planar devices, particularly within the wavelength range of λ = 400–720 nm where R in the nanostructured device is ≤5%. The decrease in reflectance can in part be attributed to an effective grading in refractive index by the tapered sub-wavelength scale structuring^28^,^29^. As a result reflectance is lower than the planar device in which interfaces have relatively abrupt changes in refractive index. The absorption spectra from the individual ITO, MoO_3_, PbS CQD, and Au layers are shown in Supp. Figure 1.

**Figure 2:**
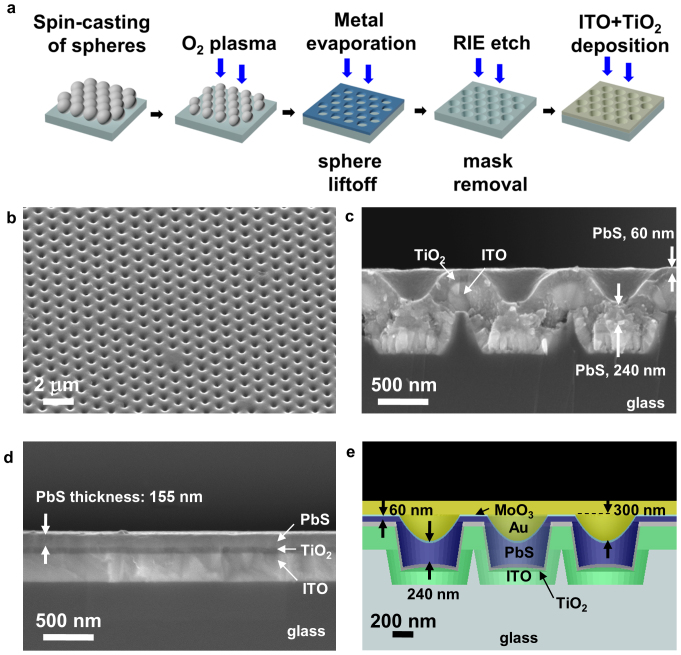
Fabricated nanostructured CQD solar cells. (a) Illustration of the fabrication process for the periodic nanostructured substrates. The periodicity (sphere size) is 1 μm. The device is completed by depositing the PbS CQDs via dip-coating and evaporation of the MoO_3_/Au/Ag top contact/mirror. (b) 45° tilt angle and (c) cross-sectional SEM images of the nanostructured CQD solar cell with PbS layer (before metal contact evaporation). (d) Cross-sectional SEM image of the planar control device using the same preparation procedure without the nanosphere lithography step. (e) Cross-sectional illustration of the corresponding FDTD simulation structure based on the cross-sectional SEM.

We prepared nanostructured substrates based on the optimized device design of Figure 1 using nanosphere lithography. The process flow for the substrate preparation is shown in Figure 2a. Borofloat glass substrates were first coated by a closely-packed monolayer of 1 μm diameter polystyrene spheres by spin-casting (process steps are described in the methods section), followed by a reduction of the sphere size by O_2_ plasma etching. Al metal evaporated through the spheres acted as the etch mask for the following glass reactive ion etch (RIE) etching step. Next, the structured substrate was coated using sputtered indium tin oxide (ITO) followed by an n-type TiO_2_ layer. The device is completed by a semi-conformal PbS layer prepared by dip-coating, followed by top contact (MoO_3_/Au/Ag) evaporation. SEM images (45° tilt angle and cross-sectional) of the nanostructured CQD solar cell with PbS CQDs, but before top metal contact evaporation are shown in Figure 2b and 2c. From microscope images, 90% of the active device area was calculated to be nanostructured. For comparison, a cross-sectional SEM image of the reference planar device prepared under the same conditions as the structured substrate is shown in Figure 2d. The PbS film thickness is 180 nm. A model structure based on the cross-sectional SEM image is shown in Figure 2e. The main difference between the modelled structure (Figure 1a) and the fabricated devices (Figure 2c) resides in the conformality of the PbS CQD layer: the deposited PbS film is very thin at the top of the structures (thickness = 60 nm) and thick within the trenches (thickness = 240 nm), caused by capillary forces during the layer-by-layer quantum dot film deposition process. Furthermore, the PbS CQD film does not contour to the sharp edges of the substrate and is rounded on the top. In addition to the non-conformal PbS film, the ITO is also not completely flat at the bottom of each trench, and is slightly thicker at the centre of the trench than edge, which is caused by shadowing during deposition^30^.

**Figure 3:**
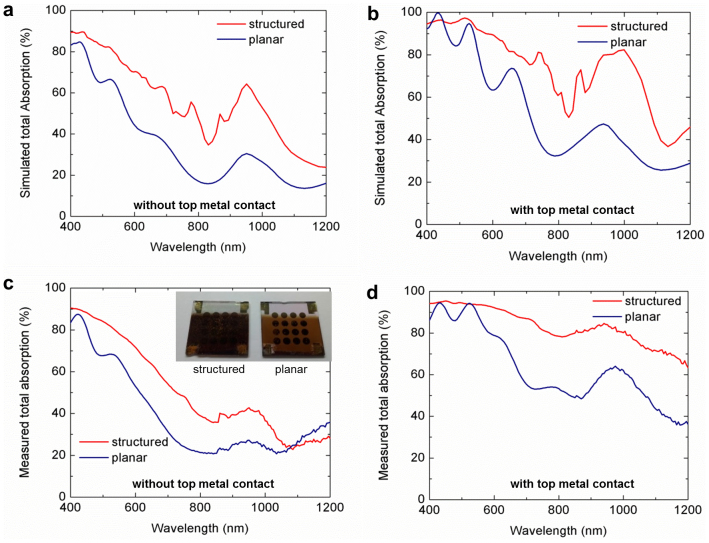
Optical properties of the nanostructured CQD solar cell. FDTD simulations of the total absorption in the CQD solar cell using the model shown in Figure 2e (a) without top metal contact and (b) with top metal contact. Total absorption measurements (c) without top metal contact and (d) with top metal contact. The inset of c shows a photograph of the structured and planar device viewed from the glass-side of the substrate.

We refined the simulation model to account for the more realistic device stucture revealed by the SEMs of Figure 2. The results of the FDTD simulations of the total absorption are shown in Figure 3a without the top metal contact and Figure 3b with the top metal contact for the structure shown in Figure 2e. The corresponding experimental measurements of total absorption (Methods section *Absorption measurements*) are shown in Figure 3c without the top metal contact and Figure 3d with the top metal contact. Taking into consideration non-idealities in periodicity, including incomplete coverage of spheres and missing spheres (Figure 1b), and cracking in the PbS film, the experimental absorption measurements agree reasonably well with the FDTD simulations. Both with and without the top metal contact, the structured device shows strong broadband absorption enhancement compared to the planar film. The volume of the PbS film (shown in Figure 2c) calculated by integrating the mesh volume of the PbS region in the 3D simulation structure (Figure 2e) is equivalent to a 163 nm thick planar film, which is less than half of the thickness usually used in the highest performing planar devices. The volume of the structured device agrees to within 7% of the planar control film (thickness = 155 nm).

**Figure 4:**
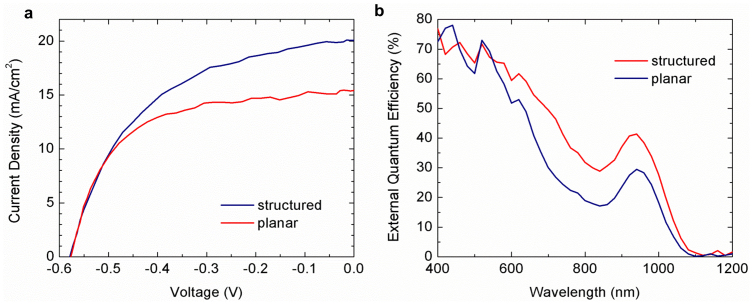
Measured nanostructured CQD device properties. (a) The current density vs. voltage curves under simulated AM 1.5 illumination for planar and structured solar cells. The short-circuit current density (*J_sc_*) of the nanostructured device is 20.2 mA/cm^2^, a 31% improvement compared to the planar control (15.4 mA/cm^2^). The PbS volume is approximately equal in both devices. (b) The external quantum efficiency (EQE) spectra for planar and structured solar cells. Most of the EQE improvement occurs within the wavelength range of λ = 600–1100 nm which agrees with the absorption measurements in Figure 3d.

The current density (*J*) vs. voltage (*V*) curves under simulated AM 1.5 illumination of the nanostructured and planar CQD solar cells are shown in Figure 4a. The structured device exhibited noticeable improvement in short circuit current (*J_sc_*) of 20.2 mA/cm^2^ versus 15.4 mA/cm^2^ for the planar device, corresponding to an improvement of 31%. The open-circuit voltage is the same (0.57 V) and the fill factor of the structured device was 51.6% versus 60.2% for the planar device. The drop in fill factor can be attributed to cracking in the PbS film and less-than optimal coverage of the top MoO_3_/Au contact on structured devices since both the MoO_3_ and Au layers were deposited by a line-of sight evaporation deposition technique. Overall, the photovoltaic conversion efficiency (PCE) of the structured device was 6.0% versus 5.3% for the planar device. In both the nanostructured and planar devices, the volume of CQDs is less than half of that used in record performance devices, which typically have film thicknesses in the range of 350–400 nm^5^,^8^,^24^. The external quantum efficiency (EQE) spectra, shown in Figure 4b, show that the structured device has improved charge collection over the wavelength range λ = 600–1100 nm which agrees with the absorption measurements in Figure 3d.

## Discussion

As shown in the cross-sectional SEM image in Figure 2c the thickness of the PbS film is much thicker within the trench (240 nm) than at the top (60 nm). This non-uniformity in film thickness is the main limitation in overall device performance. Making the PbS film thicker leads to an even larger nonuniformity in film thickness, leading to poor carrier transport in the thick portions of the PbS film. In addition, the Au back-reflector becomes more planarized as the number of layers is increased, reducing the optical benefit.

Future efforts will therefore benefit from realization of a conformal, stress-free semiconductor film deposition technology for CQDs. Possible deposition techniques for preparing such a film may include a dip-coating process using a thin wetting hydrophobic sidewall^31^, a spray-coating method, or electrophoresis. Alternatively, vacuum process deposition methods (e.g. atomic-layer deposition or chemical vapor deposition) of semiconductor films could also lead to conformal layers.

In conclusion, sub-wavelength sized structured substrates for bottom-illuminated solar cells demonstrate broadband absorption enhancement while maintaining short collection distances for photogenerated carriers. FDTD simulations show that absorption gain is due to enhancement in electric field intensity particularly near the surface of the structured Au contact. Although structured substrates were prepared by nanosphere lithography as a proof of concept in this work, such structuring can also be prepared by other techniques that can be scaled up to large areas such as the nano-imprint technique or interference lithography. As such, the structural and optical design discussed in this work is a viable pathway for better utilizing the full solar spectrum in CQD solar cells.

## Methods

### Colloidal quantum dot synthesis

The PbS CQDs were synthesized based on a previously published method^32^ that incorporated an in-synthesis solution-phase metal halide (CdCl_2_) treatment step^2^. 1.0 mL of metal halide precursor (CdCl_2_ and tetradecylphosphonic acid (TDPA) dissolved in oleylamine at a 13.6:1 Cd:TDPA molar ratio) was added to the reaction flask after injection of the sulfur source and when the temperature reached 60°C during the cooling process. Nanocrystals were isolated by adding acetone and centrifugation when the temperature reached 30–35°C. CQDs were purified by dispersion in toluene, reprecipitation in 1:1 acetone:methanol and redisolved in anhydrous toluene. Finally, the solution was washed in methanol three times and redispersed in octane at a concentration of 50 mg/mL.

### Finite-difference time-domain simulations

Finite-difference time-domain simulations were carried out using Lumerical FDTD Solutions software (http://www.Lumerical.com) version 8.5. All simulations were for a hexagonal array of three dimensional structures (periodicity = 1 μm) with periodic boundary conditions in the x and y directions. A broadband (λ = 400–1200 nm) planewave source polarized along the y-axis (axes labeled in Figure 1a) was incident from within the glass region. The absorption in each material was calculated by integrating the absorption only of matching refractive index of a particular material.

### Nanostructured substrate preparation

The process flow of the nanostructured substrate preparation is illustrated in Figure 2a. All devices were prepared on Borofloat glass (25 mm × 25 mm × 1.1 mm). The glass substrates were first treated to make the surface hydrophilic^33^. Substrates were ultrasonicated while immersed in an acetone bath for 10 min, followed by a de-ionized (DI) water rinse. They were then treated in piranha solution (1:3 ratio of H_2_O_2_:H_2_SO_4_) at a temperature of 80°C for 30 min under constant stirring followed by RCA-1 cleaning (1:1:5 ratio of NH_4_OH:H_2_O_2_:DI H_2_O) at a temperature of 80°C for 30 min. The substrates were rinsed in DI H_2_O and used immediately after N_2_ drying. 45 μL of polystyrene spheres (1 μm diameter, 10% wt. dispersion in H_2_O, purchased from Alfa Aesar) were spin coated using a modified two step method^34^: 1) 500 RPM for 10 sec., 2) 700 RPM for 120 sec. The size of the spheres were tuned by O_2_ plasma etching (Trion Phantom II RIE/ICP System). The O_2_ flow rate was 10 standard cubic centimeters per minute (sccm), the pressure was 30 mTorr, and the RF power was 120 Watts. An aluminum etch mask (60 nm in thickness) was then deposited by thermal evaporation (BOC Edwards Auto 306 thermal evaporator). Polystyrene spheres were lifted-off by ultrasonic agitation in a dichloromethane bath solution for 1 hour. The glass substrates were etched by Reactive-Ion-etching (Trion Phantom II RIE/ICP System) using CHF_3_/O_2_ gas. The CHF_3_ and O_2_ flow rates were 20 sccm and 5 sccm, respectively, the pressure was 30 mTorr, the RF power was 250 Watts, and the ICP power was 300 Watts. The etch duration was 125 seconds, which resulted in an etch depth of 400 nm. The aluminum film was removed by wet etching in chrominum etchant heated to 80°C. The bottom contact was deposited by magnetron sputtering (Angstrom Engineering Åmod deposition system in an Innovative Technology glovebox) using a 3” diameter Indium-Tin-Oxide (In_2_O_3_/SnO_2_, 90/10 wt%) target. Substrates were heated to 380°C and rotated during deposition, and the film thickness was 260 nm on a planar substrate. The TiO_2_ layer was deposited by magnetron sputtering from a 3” diameter TiO_2_ (99.9% purity) target. The film thickness was 50 nm on a planar substrate. The TiO_2_ was treated with a 120 mM TiCl_4_ solution at 70°C for 30 min, followed by an anneal at 400°C for 60 min in air ambient.

### CQD device fabrication

The semi-conformal PbS film was prepared by a layer-by-layer (LBL) dip-coating method. Each layer consisted of four solution immersion steps: 1) dilute mercaptopropionic acid (MPA) (0.02% in methanol), 2) colloidal PbS quantum dots (7.5 mg/mL in hexane), 3) MPA (0.2% in methanol), 4) methanol wash. Each solution was kept in a Pyrex beaker and had a volume of 25 mL, except the CQD beaker which had a volume of 16.5 mL. After each step the substrates were dried in air for 3 min except the second MPA step, which was dried for 2 min. A slow flow of compressed air was used to control the relative humidity to about 30%. Note that the drying time and humidity had a strong influence on film quality and thickness. Both planar and nanostructured substrates were coated simultaneously with a total of 12 layers. The top electrode consisted of 25 nm of thermally evaporated MoO_3_, followed by 150 nm of e-beam evaporated Au, and 240 nm of thermally evaporated Ag. These layers were deposited using a base-pressure of <8 × 10^−7^ Torr in an Angstrom Engineering Åmod deposition system in an Innovative Technology glovebox. The contact size was 0.049 cm^2^.

### Absorption measurements

All absorption measurements were done using a Perkin Elmer Lambda 950 UV-Vis-NIR spectrophotometer equipped with an integrating sphere. Samples were place at the centre of the integrating sphere tilted at an angle of 20° relative to the incident beam. The total transmission (T) and reflectance (R) were collected by the integrating sphere detector with all ports closed except that for the incident beam. Absorption was calculated as 100% -T-R. The 100% transmission baseline measurement was an empty sphere.

### AM1.5 photovoltaic device characterization

All photovoltaic and EQE measurements were carried out under N_2_-flow. Current density – voltage curves were measured using a Keithley 2400 source meter with illumination from a Solar Light XPS 200 solar simulator with an irradiance of 100 mW/cm^2^. The active area of the solar cell was illuminated through a circular aperture with an area of 0.049 cm^2^. The power was measured using a Melles-Griot broadband power meter. The spectral mismatch between measured and actual solar spectral performance was estimated using a calibrated reference solar cell from Newport. A total spectral mismatch of ~5% was taken into account by applying a multiplicative factor of 0.95 to measured current density values. The uncertainty of AM1.5 measurements was estimated to be ±7%.

### External quantum efficiency measurements

External quantum efficiency spectra were measured under monochromatic light (400 W xenon lamp source passing through a monochromator with order-sorting filters) which was chopped at 220 kHz. A constant 1 sun intensity white-light source simultaneously illuminated the device during measurements. The monochromatic light power was measured using Newport 818-UV and Newport 818-IR power meters. The current response was measured using a Stanford Research Systems lock-in amplifer at short-circuit conditions. The uncertainty of the EQE measurements, calculated by taking the root-mean-square error of all equipment used and variation in pixel area, was ±3%.

## Author Contributions

M.M.A. designed, fabricated, and characterized devices and performed FDTD simulations with guidance from S.M.T. and E.H.S. A.J.L. aided in developing experimental methods. A.J.L. and X. L. developed the dip coating method. S.H. assisted with experimental design. M.M.A. wrote the manuscript. All authors contributed to editing and reviewing the manuscript.

## Supplementary Material

Supplementary Information Supplementary Information
