# Supplementary material for: Broadband solar absorption enhancement via periodic nanostructuring of electrodes
Source: Sci Rep. 2013 Oct 14;3:2928. doi: 10.1038/srep02928 (PMC3796292; doi:10.1038/srep02928)
Supplement: Supplementary Information [file srep02928-s1.pdf]

Supplementary Information for:

Broadband solar absorption enhancement via periodic nanostructuring of electrodes

Michael M. Adachi, André J. Labelle, Susanna M. Thon, Xinzheng Lan, Sjoerd Hoogland, Edward H. Sargent

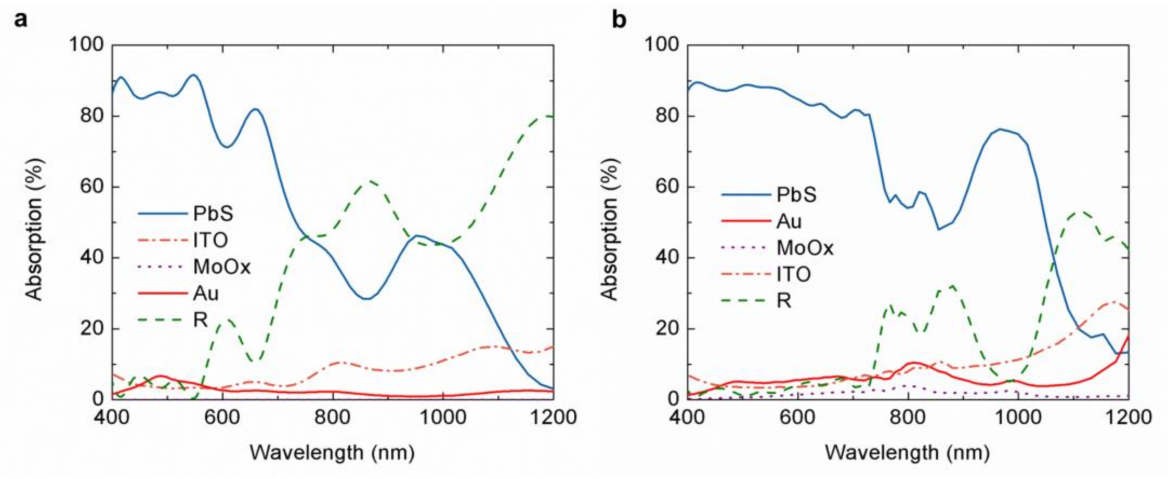

Figure S1. FDTD simulations of absorption by different layers (PbS, ITO, MoO_3, Au) and reflectance (R) in the planar (thickness=290nm) device (a) and the structured device (b) shown in Figure 1a.
